# Multilevel Contiguous Osteoporotic Lumbar Compression Fractures: The Relationship of Scoliosis to the Development of Cascading Fractures

**DOI:** 10.7759/cureus.1962

**Published:** 2017-12-19

**Authors:** Alex Sabo, Jesse Hatgis, Michelle Granville, Robert E Jacobson

**Affiliations:** 1 Neurology, Pain Management, Nova Southeast/larkin Community Hospital; 2 Pain Management, Phoenix Neurological and Pain Institute; 3 Miami Neurosurgical Center, University of Miami Hospital

**Keywords:** osteoporotic compression fractures, multiple compression fractures, lumbar compression fractures, cascading osteoporotic fractures, sacral insufficiency fracture, vertebral augmentation, kyphoplasty, lumbar degenerative scoliosis, lumbar scoliosis, adjacent segment osteoporotic fractures

## Abstract

Osteoporotic patients can present with either single or multiple fractures secondary to repeated falls and progressive osteoporosis. Multiple fractures often lead to additional spinal deformity and are a sign of more severe osteoporosis. In the thoracic spine, multiple fractures are associated with the development of gradual thoracic kyphosis but neurologic deficits are uncommon. In the lumbar spine, patients with multiple lumbar fractures have more constant lumbar pain, may have symptoms related to concurrent lumbar stenosis or degenerative scoliosis, and may present with radiculopathy, especially with fractures at L4 and L5. In a review of a series of patients with recurrent multiple lumbar fractures or 'cascading' fractures, it was found that all the patients were female, had severe osteoporosis, often untreated, had a previous history of multiple previous thoracic and lumbar fractures, and all had associated scoliotic spinal deformities ranging from 6^o ^to 50^o^. It was found that if the curve progressed and the greater the degree of curvature, the more frequently subsequent multiple fractures developed, leading to recurrent acute episodes of pain. Forty percent also had additional sacral insufficiency fractures, an unusually high percentage. Biomechanically, the lumbar spine is both more mobile and supports a larger portion of the spinal load compared to the thoracic spine. The existence or worsening of a lumbar spinal deformity from degenerative lumbar scoliosis shifts the mechanical forces more to one side on already weakened osteoporotic lumbar vertebrae and sacrum, leading to an increased incidence of these fractures. Because of the chronic and uneven lower lumbar spinal load with severe vertebral osteoporosis in certain patients with repeat lumbar fractures and worsening degenerative lumbar scoliosis, there may be a rationale to add preventive vertebroplasty at adjacent vertebral endplates when treating acute recurrent lumbar fractures to decrease the incidence of recurrence in other vertebrae.

## Introduction and background

Osteoporotic vertebral compression fractures (VCF) are distributed throughout the thoracic and lumbar spine in a biphasic distribution, with a peak between T6 and T9 and between T11 and L2. These levels account for 75% to 80% of all vertebral fractures. Lower lumbar and sacral fractures are much less common, making up less than 15% combined in large series [1-2]. Radiological studies demonstrate that thoracic fractures are more typically anteriorly wedge-shaped, leading to kyphosis of the individual vertebra. Thoracic kyphosis can also develop without definitive cortical fractures because of gradual and subtle loss of anterior cancellous bone height involving multiple vertebrae. Lumbar fractures typically show mid-vertebral endplate collapse but without severe kyphosis and often have biconcave defects centered in the middle of the vertebra [3]. Many patients with single fractures respond to conservative treatment but persistent pain or the development of deformity may require intervention with either vertebral augmentation or kyphoplasty. There is a five-fold increased incidence of other vertebral and osteoporotic fractures after the diagnosis of the initial fracture, so continued follow-up and medical treatment for the osteoporosis is essential [1]. When the patient is initially evaluated, it is common to detect previous fractures, so determining the age of the fractures is often critical in planning treatment. The age of the fracture can be determined by signs of bone marrow edema on magnetic resonance imaging (MRI) or marked isotope uptake on bone scans [4]. Later development of additional osteoporotic fractures is common with or without clear evidence of trauma. Additional new fractures can be due to the progression of the underlying osteoporosis, especially if the bone mineral density is very poor and the patient is noncompliant with treatment, has another fall or injury, or as a result of secondary effects from spinal deformity and the effects of vertebral augmentation or kyphoplasty on an adjacent vertebra [4].

Lower lumbar vertebral compression fractures are less common. Lower lumbar fractures from L3 to L5 compose only 8% to 12% of a large series of VCF and sacral fractures less than 4% of large study groups [5-6]. Generally, VCFs are more commonly associated with localized spinal pain than with neurologic complaints; however, lower lumbar fractures, especially at L5, have a significant incidence of presenting with radiculopathy. Leg pain is seen in 15% to 25% of lumbar fractures and is definitely more frequent with fractures at L4 and L5 compared to L2 and L3 [5-6]. Since these elderly patients frequently have lumbar stenosis (LS), often with degenerative spondylolisthesis (DS) or lumbar degenerative scoliosis (LDS), determining the cause of radiculopathy or directly relating it to the fracture can be difficult [6]. When a patient develops subsequent fractures after treatment with either vertebroplasty or kyphoplasty, it is important to differentiate between the worsening of a previously treated fracture and the development of a new fracture at an adjacent or another spinal level [7]. The reported frequency of the development of symptomatic new fractures at an adjacent level after previous vertebroplasty or kyphoplasty ranges from 10% to 29% [8]. Adjacent level fractures have been attributed to increased vertebral rigidity from the cement in the treated osteoporotic fracture and to change in sagittal balance, so with residual compression, the superior vertebra is affected by more anterior loading [7-8]. Biomechanical studies have shown that the volume and location of the cement have only a minimal direct effect on the adjacent vertebra. Leakage of cement into the adjacent disc space, excessive cement injection into the fracture site and more unilateral placement of the cement from the initial vertebroplasty or balloon kyphoplasty does not statistically effect either pain relief or further collapse but can have some effect on the adjacent vertebra [9]. However, cement placement in the cancellous bone closer to the fractured and collapsing endplate seems to be beneficial in preventing further collapse or adjacent segment collapse [10]. Experimental studies demonstrated that load-bearing forces may be the predominant reason for the development of adjacent level fractures. Anterior kyphotic spinal deformity developing from the fractured vertebra causes angulation, which leads to increased downward pressure on the anterior cancellous bone of the adjacent superior vertebrae. This is confirmed with studies showing that over 70% of the thoracic kyphotic deformity is due to the wedge collapse of just one vertebra but is associated with progressive kyphotic deformity over multiple segments secondary to softening and anterior height decrease without a clear fracture of the vertebrae [11]. In the lumbar spine, kyphosis is infrequent and radiologic studies show that both lateral shifting and mild degrees of scoliosis are often a result of uneven degenerative disc space collapse and lateral vertebral collapse. In the lumbar spine, the biomechanical effect of degenerative scoliosis alters load bearing in both the sagittal and axial plane and is associated with a loss of the normal lumbar lordosis rather than kyphosis [6]. The loss of lordosis combined with lateral angulation shifts the upper body load more laterally than anteriorly onto the inferior adjacent vertebra. This partially explains the finding that lumbar adjacent level fractures often affect both the superior and inferior vertebrae [12]. Sagittal deformities, such as spondylolisthesis and the lumbar-sacral inclination, have also been associated with an increased incidence of lumbar fractures but, more typically in these cases, the fractures are found in the vertebra superior to the deformity since these deformities commonly occur at either the L4-5 or L5-S1 spinal level [13-14].

**Figure 1:**
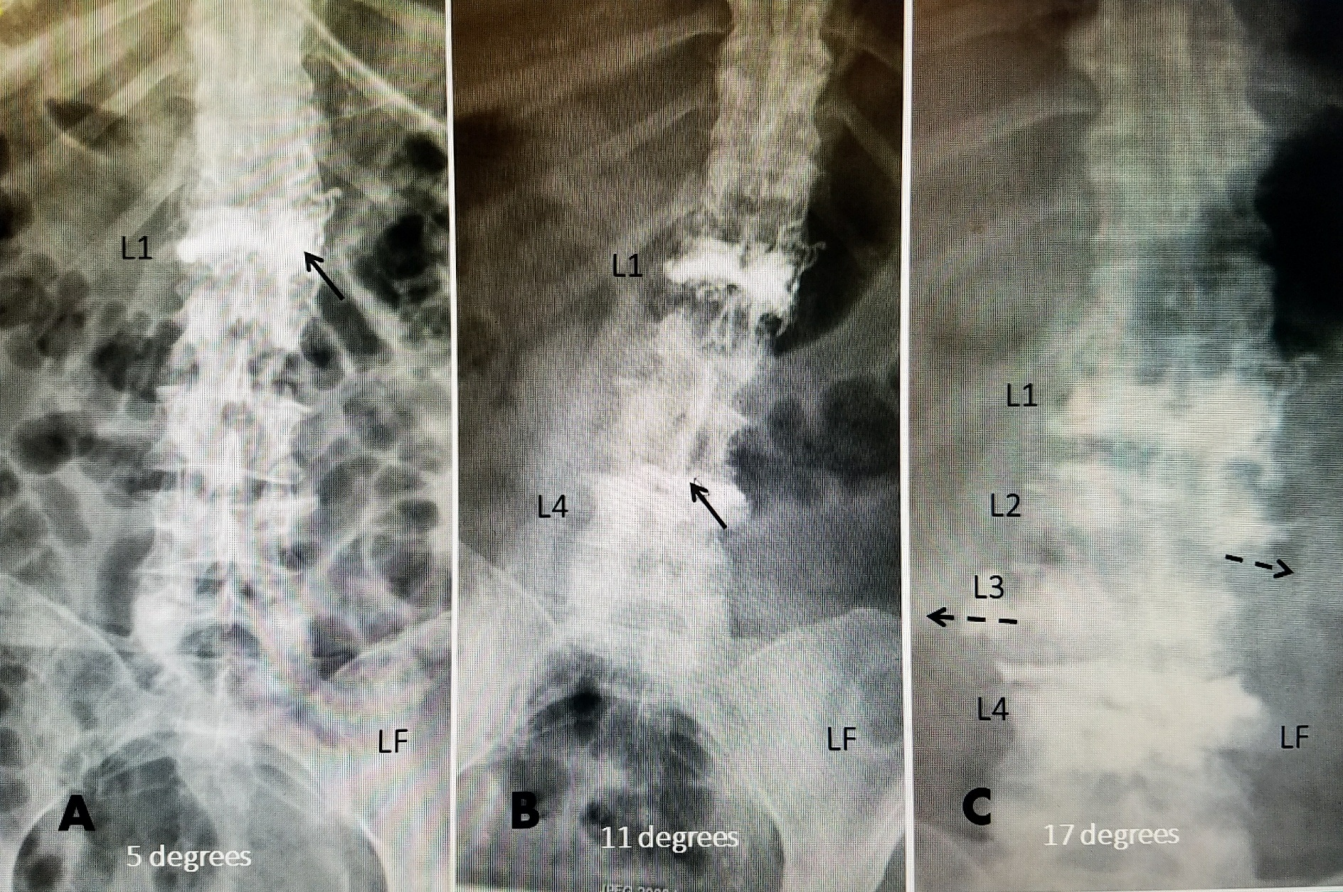
Progressive degenerative scoliotic deformity from multiple lumbar compression fractures The patient is a 63-year-old female on chronic steroids for Sjogren's syndrome. She was taking alendronate 70 mg before the initial fracture. Her bone mineral density on medical therapy was -2.9. A: Initial radiograph showing L1 vertebral kyphoplasty (black arrow) and mild 3^o ^angulation to the left at L3 and slightly to the right at L1-2. B: Nine months later, after a pain-free interval, she had another minor fall. She developed a second fracture at L4, treated with vertebral augmentation with polymethyl-methacrylate (PMMA) (black arrow). There now is an 11^o^ angulation. She was placed on a teriparatide 20 ugm daily injection. She had residual low-grade back pain after the second kyphoplasty but was fully active. C: Eighteen months later, the patient started to complain of more constant back pain without a specific fall. Studies showed a worsening of the lateral scoliosis with the coronal angulation increased to 17^o^. A bone scan showed new fractures at L2 and L3. She was treated with a two-level vertebroplasty and vertebral augmentation with Cortoss (Stryker Corporation, Kalamazoo, Michigan, USA), had a residual dull lumbar pain, and was advised to use a lumbar sacral orthosis (LSO).

Pre-existing scoliosis was identified as a risk factor for osteoporotic fractures in women and found in up to 48% of study groups [3]. However, it is important to note that these studies were done before vertebroplasty and kyphoplasty procedures were introduced, so the relationship between these minimally invasive percutaneous cement procedures and the effects on pre-existing lumbar scoliotic curves has not been studied in detail. Epidemiologic studies of DLS show that asymmetric disc collapse and lumbar curvature is a common deformity of the aging spine. Degenerative lumbar scoliosis is much more frequent in elderly females, the same population at higher risk for osteoporosis and VCF [15-16]. Lumbar stenosis is frequently found concurrent with degenerative scoliosis and the resulting altered biomechanics over the long term worsens the facet degeneration [6]. Curves of 10^o^ or more followed for over 12 years in longitudinal studies have at least a 20% chance to continue to progress [3,16]. Chronic idiopathic curves in elderly patients are more static but tend to have osteoarthritic facet hypertrophy that adds a degree of rigidity as well as localized stenosis and can be associated with an increased incidence of upper lumbar fractures [3,15]. The literature does not emphasize the role of lateral spinal deformity and the effects of abnormal biomechanical loads that contribute to recurrent or multiple lumbar fractures after vertebral augmentation or kyphoplasty. This review examines patients that developed multiple adjacent or sequential osteoporotic lumbar fractures with underlying scoliosis. These sequential fractures have been described as cascading osteoporotic fractures in case reports [17]. The group of patients reviewed for this report all had multiple lumbar fractures and underlying degenerative lumbar scoliosis or idiopathic thoracic-lumbar scoliosis and went on to develop cascading lumbar fractures requiring multilevel repeat vertebroplasties for pain control (Figure 1).

## Review

Degenerative lumbar scoliosis (DLS) is seen predominately in older females [3,15]. Long-term longitudinal studies found that even as early as a mean age of 54, 29% of the females already had lumbar curves greater than 5%. In a 12-year follow-up study, while some of these mild curves resolved, another 29% developed progressive curves greater than 10%, especially when followed into the post-menopausal osteoporotic age group when they also were at higher risk for VCF [16]. To examine this inter-relationship in more detail, the charts of all cases undergoing vertebroplasty and vertebral augmentation or kyphoplasty over a 36-month period were re-examined for the development of successive recurrent lumbar fractures or 'cascading' fractures or scoliosis [17]. Patients that were found to have multiple contiguous lumbar fractures were reviewed regarding the associated spinal deformity, such as scoliosis, spondylolisthesis, and levels of fractures (Table 1).

**Table 1:** Sex, age, first fracture, subsequent fracture, previous surgery, orthopedic fracture, and other factors FX: fracture; SUB: subsequent fracture; SX: surgery; THOR: thoracic; ORTHO: knee or hip orthopedic fracture * Less than 10 degrees ** 10-20 degrees *** Greater than 20 degrees ^ Chronic steroid use

SEX	AGE	1^ST^ FX	SUB FX	SACRUM	SCOLIOSIS	SX	THOR FX	ORTHO FX	OTHER COMORBIDITIES
F	87	T12	L4 & L5	NO	YES*	YES	1	YES	CANCER/MULTIPLE FALLS
F	75	T8 & T9	L2, L3, & L4	YES	YES**	NO	2	YES	SJOGREN'S^/STENOSIS
F	81	T11, L1, & L3	L4 & L5	YES	YES*	YES	1	YES	DIABETES/PROLONGED BED REST
F	87	L1	L2 & L3	NO	YES***	NO	0	N0	SEVERE OSTEOPOROSIS
F	81	T9, T11, &T12	L4 & L5	NO	YES**	YES	3	N0	HYPOTHYROIDISM
F	85	T6 & T8	L1, L3, & L4	YES	YES*	NO	2	N0	COPD/SLEEP APNEA
F	77	T11	L2, L3, & L4	NO	YES***	NO	1	N0	CANCER/KYPHOSIS
F	78	L2 & L3	L4	NO	YES***	NO	0	N0	ASTHMA^

**Figure 2:**
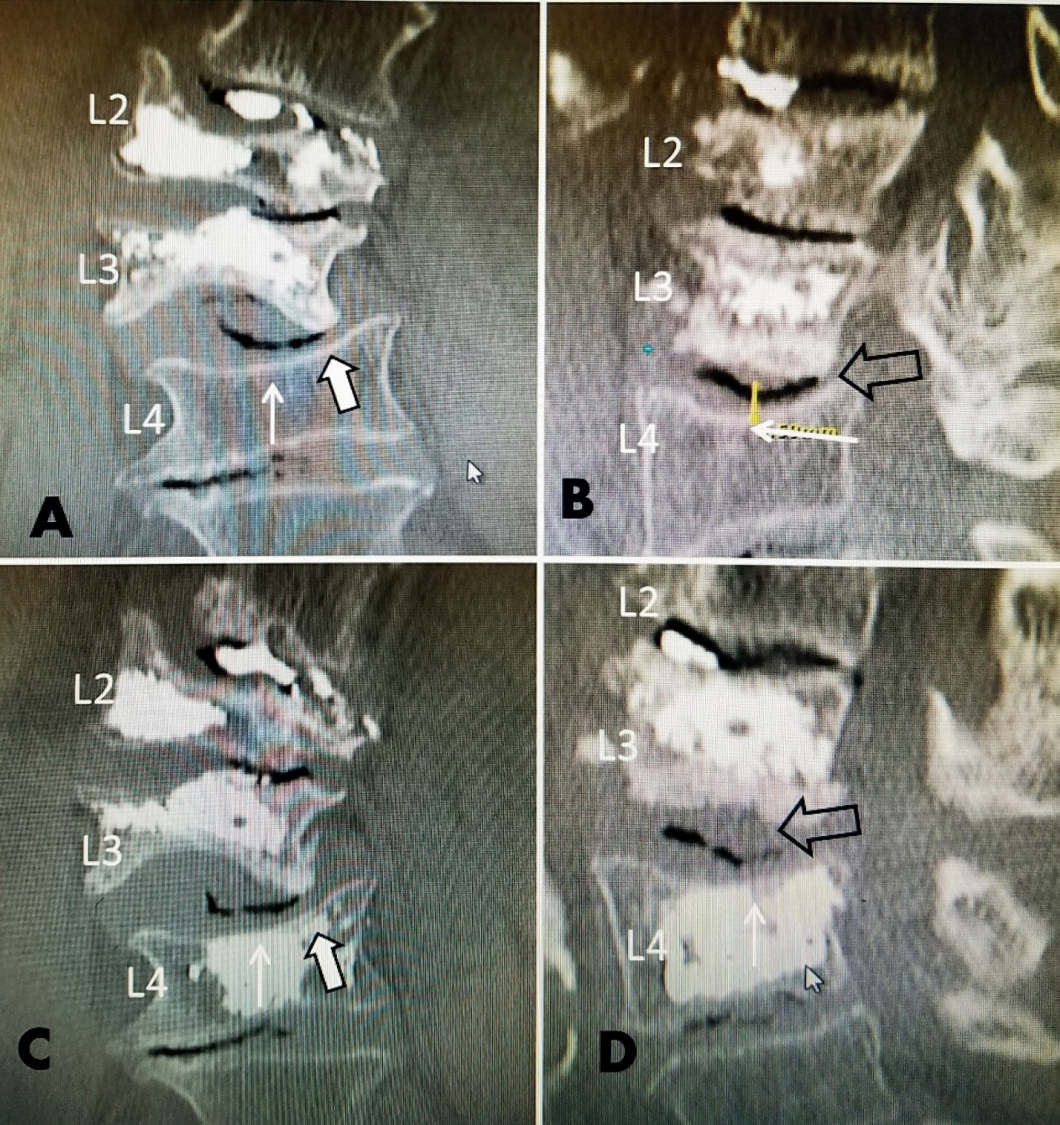
A 78-year-old female with previous kyphoplasty at L2 and L3 with progressive right convex scoliosis and a fracture at L4 A: Coronal computerized tomography (CT) showing a superior endplate collapse at L4 (solid white arrow). The edge of the inferior endplate of L3 is pressing into the depressed superior endplate of L4 before the actual development of an acute fracture (solid, large white arrow with black border). There are multiple vacuum disc defects at L2-3, L3-4, and L4-5. The angled L3 vertebra is collapsing into the superior endplate fracture of L4 (solid, large white arrow with black border). B: A lateral CT scan showing superior endplate subsidence without a clear fracture at L4 (solid white arrow). The bone scan showed mild uptake at L4. The disc space height between L3 and L4 is normal (yellow arrow). There is a large vacuum cleft sign across the entire L3-4 disc space (open black arrow). C: Coronal CT scan after kyphoplasty combined with the insertion of a Kiva polyethyletherketone implant PEEK-OPTIMA (Benvenue Medical Santa Clara, California, USA) at L4. The cement and PEEK-OPTIMA cage was placed directly under the center of L4 ( small white arrow), which is the downward pressure point from the shifted L3 vertebra (large, solid white arrow with border). D: Sagittal CT scan after L4 kyphoplasty with a Kiva system and polymethyl methacrylate cement (PMMA). The cement is filling the entire peek cage placed transpedicularly, providing strong support under the collapsing superior endplate of L3 (solid white arrow). Note that the vacuum cleft previously seen in the L3-4 disc space is much smaller (open, large black arrow). This was performed with the patient's consent as a preventive kyphoplasty because of the lateral instability and angulation of the L3 inferior vertebrae into L4 on the coronal views. The patient did not develop further fractures over a 24-month follow-up.

The review identified a total of eight patients, all female, with an average age of 81.4 years, who all had a history of multiple previous thoracic or upper lumbar fractures and scoliosis. Three patients had a minimum of three fractures and three had five fractures. Three patients, or 40%, also developed sacral insufficiency fractures. Sacral insufficiency fractures typically make up less than 3% to 5% of overall osteoporotic fractures [1-2]. Three patients also had other osteoporotic fractures in the shoulder or hip. The interval from the first treated fracture until recurrent lumbar fractures were diagnosed and treated was from one month to four years but the mean interval between recurrent fractures was only six months. Two of the patients had a history of chronic steroid use for treatment for other co-morbidities. All patients had lumbar scoliosis of varying severity but 38% had greater than 20^o^ curves. Six patients had degenerative scoliosis isolated only to the lumbar spine. These patients had progressive lateral listhesis, leading to the development of cascading lumbar fractures (Figure 2).

**Figure 3:**
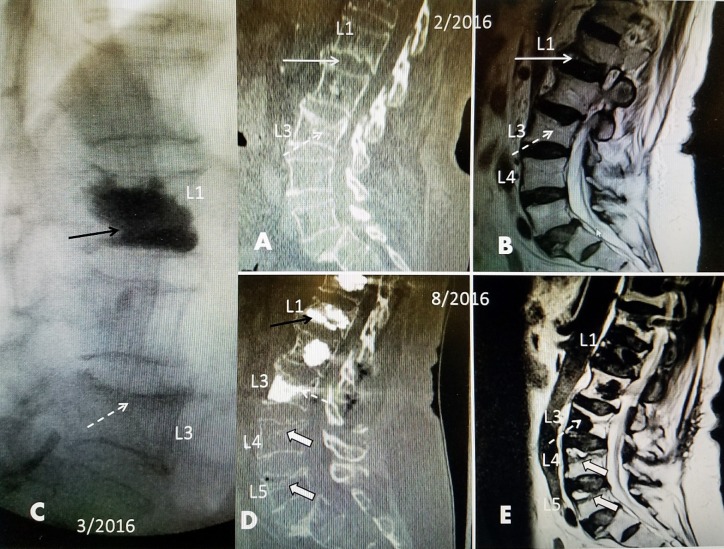
Sequential radiologic studies from an L1 fracture to cascading lumbar and sacral fractures over six months A+B: A 78-year-old, osteoporotic, diabetic female had a fall, sustaining an acute L1 inferior endplate fracture (solid white arrow). Computerized tomography (CT) and magnetic resonance imaging (MRI) showed that she had edema at L1 and an old superior endplate fracture at L3 that was not edematous on a T2 MRI scan (dashed white arrow) C: She had a kyphoplasty at L1 (solid black arrow) and L3 was not injected. D+E: Because of persistent pain and a persistent positive bone scan at L1, she was operated two months after the kyphoplasty by another surgeon. Although there was straightening without kyphosis, cortical screw instrumentation from T11 to L3 was performed. Cementing of the bicortical screws was necessary due to severe osteoporosis of the vertebral bodies. She developed chronic wound drainage and remained bed-bound on intravenous antibiotics. She started rehabilitation but then developed severe lower back pain. CT and MRI three months after the instrumentation demonstrated superior endplate fractures of both L4 and L5 (large, solid white arrows) with marked vertebral edema and intervertebral fluid clefts on the MRI scan, indicative of new acute fractures.

**Figure 4:**
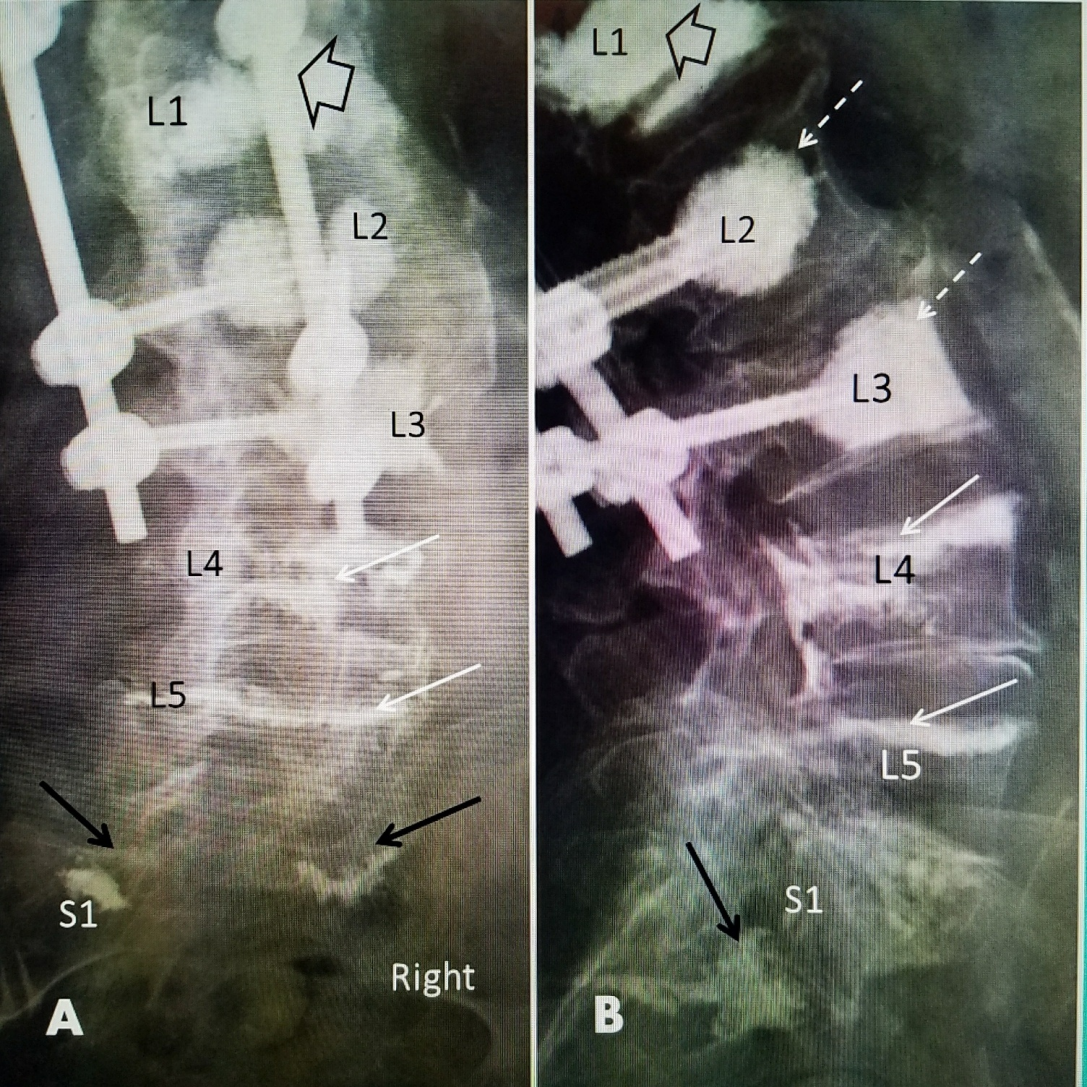
Post vertebroplasty films from patient in Figure 3 (a 78-year-old female) who had an L1 fracture and kyphoplasty followed two months later by T11 to L3 instrumentation. She went on to develop progressive cascading fractures: L4, L5, and sacrum A: Anteroposterior (AP) radiograph six months after original kyphoplasty at L1 showing 18^o^ dextroscoliosis. She had an L1 kyphoplasty (open, large black arrow) and then, two months later, T11 to L3 instrumentation with cemented bicortical screws (dashed white arrows). A bone scan showed mild uptake at L4, L5, and bilaterally in the sacrum and left ilium. Limited vertebroplasty at L4 and L5 can be seen (solid white arrows) and cement in the sacral alae (solid black arrow) B: Lateral radiograph showing that the L4 and L5 superior endplates were treated with limited preventive vertebroplasty (solid white arrows). The sacral fractures were also treated with superior placement of cement in the sacral alae (solid black arrow). Followup over 18 months did not show further progression of the L4 or L5 vertebral compression fractures.

Two patients had long-standing idiopathic thoracic-lumbar scoliosis and developed fractures in the concavity of the lower lumbar curve. Three patients had a previous spinal surgery, and in two of the three, it involved multilevel spinal fixation, one for a previous L1 fracture and the other for spinal stenosis (Figure 3, Figure 4).

All eight patients developed multiple lower lumbar fractures. More upper lumbar fractures were across the entire vertebra but the subsequent lower fractures were located primarily toward the convex side of the lumbar scoliotic curve. Single lumbar osteoporotic compression fractures only compose 6% to 15% of large studies of VCF. Sacral insufficiency fractures normally make up less than 5% of large studies and are often overlooked but were found in 40% of our small group [18]. Sacral insufficiency fractures have been shown to be related to altered biomechanics and uneven load distribution [18-19]. Recurrent fractures adjacent to previously treated vertebra occur either as a result of another fall, increased vertebral stiffness after the adjacent vertebra is treated with cement and as a result of sagittal and coronal malalignment secondary to scoliosis, which shifts the center of the spinal load to one side [18-23]. Leakage of cement into the adjacent disc space has also been related to an increased rate of adjacent level fractures [22]. The overall incidence of these adjacent fractures ranged from 13% to 28% and is found highest adjacent to previous lumbar or lower thoracic surgery with screw instrumentation as in two of our cases [23-24].

**Figure 5:**
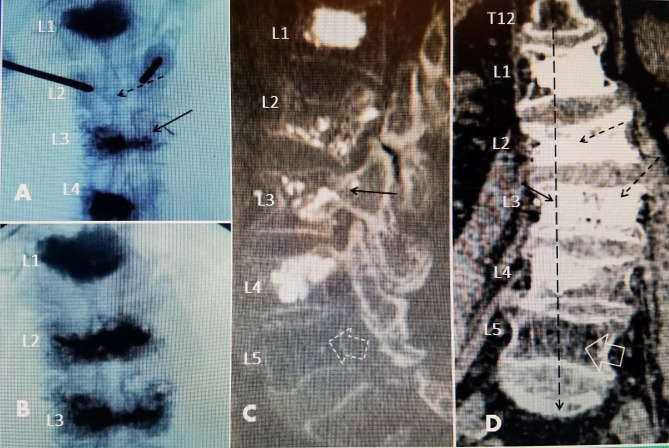
Reconstructed computerized tomography (CT) after four vertebroplasties with scoliosis A: Intra-operative fluoroscopy for L2 vertebral augmentation. The patient had redevelopment of pain with marked uptake at L2 on a bone scan (dashed black arrow) and smaller uptake L3 (solid black arrow), where insufficient cement from a previous vertebral augmentation had not completely filled the fracture. B: Postoperative film showing the filling of L2 and the additional filling of superior and lateral L3. C: Computed tomography (CT) after vertebroplasty at L3 (solid black arrow). Sagittal computerized tomography with bone window demonstrates the marked hypodensity of L5 (open, dashed, large white arrow), D: Coronal CT with soft tissue window showing complete filling of L2 and L3 ( dashed black arrows) with cement in the coronal plane. There is marked loss of bone trabecular pattern and hypodensity throughout the vertebral body of L5 (open, solid, large white arrow).The weight-bearing center of gravity relative to the lumbar scoliosis is indicated (long, dashed black arrow) passing from mid L1 to the lateral concave side of L2, L3, and L4 through the center of L5.

It is clear from this small group of patients that in the lumbar spine, loss of coronal alignment is an additional factor that can lead to uneven stress on already weakened osteoporotic vertebrae. Degenerative lumbar scoliosis is a chronic, slowly progressive deformity, so it is not likely to be able to correct the deformity with either kyphoplasty or vertebral augmentation [3,7-8]. Biomechanical studies demonstrate that the cement basically provides additional support to the cancellous bone and the adjacent endplates both for the acute symptomatic fracture and to the osteoporotic bone in the concave subjacent vertebrae [7,10,12]. When lumbar scoliosis is present and subsequently combined with cement being placed at acute or subacute symptomatic vertebrae, it can lead to cascading lumbar fractures. It is clear that there can be radiographically occult fractures only picked up on bone scan or indicated by the finding of early bone marrow edema on an MRI scan before actual collapse occurs, which is visible on plain radiographs or even CT scans [25-26]. In addition to the detection of early occult fractures with bone scans, if the patient images are carefully studied with computerized tomography and magnetic resonance imaging, it is possible to see both the shift in the center gravity line for coronal lumbar load bearing as well as the characteristic striated hypo-density of severe osteoporosis before actual fracture occurs (Figure 5).

Understanding the biomechanical importance of underlying idiopathic or degenerative lumbar scoliosis is an important step in trying to stabilize or prevent continued fracture progression [6,27-28]. Muscle imbalance and para-spinal muscle weakness and atrophy is commonly seen in post-menopausal females and can be a factor in the progression of both coronal as well as the more common sagittal spinal imbalance with degenerative lumbar scoliosis and degenerative spondylolisthesis [28]. Patients should be monitored since, in a study with over a 12-month follow-up, it was found that even with the initial restoration of height and correction of kyphosis after balloon kyphoplasty, the correction is often lost over time, causing partial recurrent vertebral collapse and deformity. Usually, even though the patient is asymptomatic with regard to pain at the treated level, they later present with adjacent vertebral fractures or progressive deformity. This progression is a result of the lateral load shift and uneven axial load bearing on weakened cancellous bone at the adjacent vertebra and sacral alae [27]. Patient comorbidities, chronic steroid use, and especially non-compliance with medication and lack of bracing are additional factors that contribute to progressive deterioration with multiple recurrent lumbar fractures [29-30]. 

**Figure 6:**
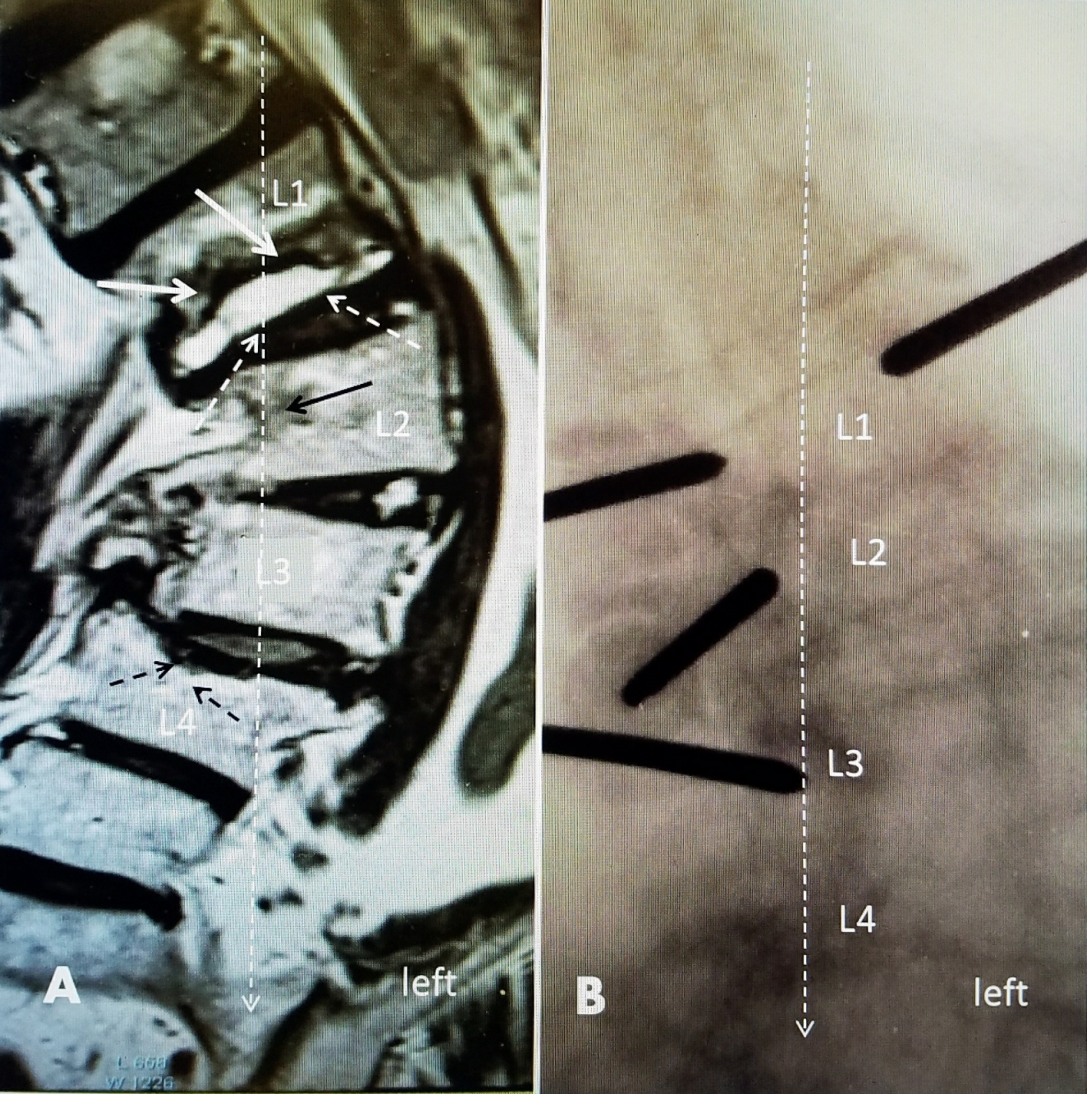
A 70-year-old female with pre-existing idiopathic scoliosis with a left convex curve of 50 degrees A: A sagittal magnetic resonance imaging (MRI) scan showing an L1 fracture with vertebral body edema of the inferior 50% of L1. There is an intervertebral fluid-filled T2 hyper-intense cleft inferiorly (white arrow). At L2 on the right, there is bone marrow edema (solid black arrow) under the L1 fracture. At L4, there is an old superior endplate fracture, without edema, and 20^o^ collapse to the center and right (dashed black arrows). B: Intraoperative image with the placement of vertebroplasty cannulas. There are two cannulas at the fractured L1. There is a single cannula in L2 and L3 in the concave part of the curve below the fracture to place cement for preventive vertebroplasty. Vertebral augmentation was performed bilaterally at L1 and unilateral cement injections at L2 and L3 were performed using Cortoss (Stryker, Kalamazoo, Michigan, USA) directly under the L1 fracture. The center line of the weight-bearing load (dashed, long white arrow) for the lower lumbar spine is seen passing through the center of L1 and the concave right side of both L2 and L3.

The development of cascading fractures in the lumbar spine often requires repeated procedures for treatment and pain relief [15,31-32]. However, the progression of the scoliotic deformity is a problem that must also be addressed and the development of multiple fractures usually causes more constant pain than seen with a single acute fracture. In high-risk patients with severe osteoporosis or as in patients with degenerative lumbar scoliosis and lumbar fractures, it is proposed to do preventive vertebroplasty at the adjacent levels when initially treating the acute fracture [33-35]. In this situation, small amounts of cement are added to the endplates above or below the acute fracture, as shown in Figure 2 [33,36]. In several, small patient studies, preventive vertebroplasty for the adjacent vertebrae above and below combined with vertebroplasty for the acutely fractured vertebra was effective in the prevention and cascading of untreated adjacent fractures. In a series of 68 patients, the control group, with kyphoplasty only at the fracture site, developed 26% adjacent level fractures while the patients received supplemental cement in the adjacent endplates had less than 3% adjacent level fractures [35]. It is important to note that these studies only evaluated sagittal compression without regard to the existence of underlying scoliosis. Targeting the adjacent endplates may reduce the necessity of multiple repeat vertebroplasty procedures [33-34]. Biomechanical studies indicate that the added cement in the region of the endplate nearest the adjacent fractured vertebra is critical to reducing the incidence of continued collapse [35]. This is of greater significance in the area of the maximum concave scoliotic curve and cement, at least, should be targeted both to the concave part of the curve and close to the endplate. In a study of technical reasons for the recurrence of a fracture within the same vertebra, it was found that one of the main reasons for the continued collapse of a previously treated vertebra was nonfilling or insufficient cement placement around the fractured area and a lack of cement specifically filling near the fractured endplate [7]. In this review, three of the eight patients, as shown in Figure 2, Figure 4, and Figure 6, had additional cement added to the superior endplate of at least one of the nonfractured vertebra adjacent to the acute fracture. None of these three patients, with a minimum 12-month follow-up, developed recurrent fractures despite a high degree of lumbar scoliosis (Figure 6).

If a patient with a lumbar VCF and lumbar degenerative scoliosis is identified with multiple potential factors that can lead to progressive lumbar fractures, such as severe osteoporosis in elderly females not on medication for osteoporosis or previous spinal surgery especially with instrumentation, there should be a higher level of concern for the development of cascading fractures as well as sacral insufficiency fractures. This is especially critical when there is a lumbar scoliotic curve greater than 10^o ^and a fracture close to the concave part of scoliosis. Treatment options vary since these patients are elderly and most have medical co-morbidities with severe osteoporosis. In some of these patients, there may be a role for preventive vertebroplasty to decrease the chance of future cascading fractures [36-38]. If the lumbar curve continues to progress and especially if there is radiculopathy associated with lumbar stenosis or degenerative spondylolisthesis, decompression with multilevel screw fixation is an option [30]. However, since the vertebrae in these patients are severely osteoporotic, over multiple levels, screw fixation may need to include supplemental bone cement and the use of multilevel bicortical screws that avoid the cancellous bone and take purchase on the vertebral endplate cortex [39]. The strongest possible medication to treat the underlying osteoporosis tolerated by the patient and supplemental bracing should always be considered with evidence of repetitive multiple lumbar fractures.

## Conclusions

Multiple osteoporotic vertebral compression fractures in the lumbar spine occur in less than 6% of cases. These fractures are frequently associated with underlying lumbar degenerative scoliosis or the development of worsening scoliosis after the lateral collapse of the initial fractured lumbar vertebra. These patients also have a higher incidence of concurrent sacral insufficiency fractures. This combination is seen most frequently in older, severely osteoporotic females, many with co-morbidities such as chronic steroid use, diabetes, and the inability to take osteoporotic medication. Many of these patients have associated spinal stenosis or degenerative spondylolisthesis that complicates their management since it is not uncommon for these patients to also have radiculopathy with lower lumbar fractures, especially at L4 and L5. Weakened muscles in older females combined with the altered spinal biomechanics because of the lateral shifting of the lumbar spinal load from the degenerative lumbar scoliosis makes the unfractured osteoporotic lower lumbar vertebrae and sacrum particularly vulnerable to the development of progressive or cascading fractures. In the lumbar spine, this is associated more frequently with coronal imbalance and deformity rather than sagittal deformity, which is commonly seen in the thoracic spine and thoracic-lumbar junction. Previous vertebral augmentation in the lumbar spine is another factor contributing to the development of progressive recurrent fractures in patients with underlying degenerative lumbar scoliosis. The underlying lumbar mechanical imbalance due to the scoliosis is a factor in the development of subsequent lumbar fractures. The unique characteristics of these lumbar fractures and the high risk of progression make it necessary to often perform multiple vertebroplasty or kyphoplasty procedures. Avoiding the progression of the fractures is a critical part of the management of these patients. A recognition of the significance of pre-existing degenerative lumbar scoliosis, aggressive medical treatment of the osteoporosis, and considering continuing lumbar bracing even after vertebroplasty or kyphoplasty all play a role. In some patients, preventive vertebroplasty in the adjacent unfractured vertebrae has been reported to be effective, as shown in these cases, and may be an option.
